# Results from adding recombinant LH for assisted reproductive technology treatment: A randomized control trial

**Published:** 2014-02

**Authors:** Mohammad-Hossein Razi, Fereshteh Mohseni, Razieh Dehghani Firouzabadi, Sima Janati, Nahid Yari, Sahabeh Etebary

**Affiliations:** 1 *Research and Clinical Center for Infertility, Shahid Sadoughi University of Medical Sciences, Yazd, Iran.*; 2 *Department of Obstetrics and Gynecology, Dezful University of Medical Sciences, Dezful, Iran.*

**Keywords:** *Assisted reproductive technology*, *Ovarian stimulation*, *Gonadotropin stimulation*

## Abstract

**Background:** Based on classical two-cell, two-gonadotropin theory, in the follicle, follicle-stimulating hormone (FSH) and luteinizing hormone (LH) put on their main effects on the granulosa and theca cells. LH is essential for androgens production. Androgens are used for estradiol production by granulosa cells. Profound suppression of LH concentrations in some normogonadotropic patients can cause several adverse effects.

**Objective: **The main clinical purpose of this study was that normoresponder women treated with controlled ovarian super ovulation for IVF or ICSI may benefit from co-administration of rLH.

**Materials and Methods:** 40 patients who were candidates for assisted reproductive technology (ART) were randomly selected. In all patients long luteal protocol was used for ovulation induction. Patients were randomly divided into two groups: Group 1 (n=20) with standard long protocol (GnRH agonist) and r-FSH alone, Group 2 (n=20) with standard long protocol (GnRH agonist) and r-FSH with r-LH. Results were statistically analyzed and compared in two groups.

**Results:** The number of retrieved oocytes, mature oocytes, cleaved embryos, transferred embryos, estradiol levels in Human chorionic gonadotropin (hCG) administration day, implantation rate and clinical pregnancy rate in group 2 were higher but not significantly different.

**Conclusion:** Administration of rLH in late follicular phase had no beneficial effect on outcomes in young women with mean age of 31 years. Maybe a greater sample size should be used to see the effects more accurately; also it is possible that rLH will be useful in older patients.

**Registration ID in IRCT:** IRCT201304302575N4

## Introduction

Development of follicles is a complicated process affected by a variety of hormones, peptides and cytokines produced both in situ and at remote areas. Based on classical two-cell, two-gonadotropin theory, in the follicle, follicle-stimulating hormone (FSH) and luteinizing hormone (LH) put on their main effects on the granulosa and theca cells (1, 2). LH is essential for androgens production. During the middle-late follicular phase androgens as precursor, are used for estradiol production by granulosa cells under aromatase activity (2, 3). Development of ovarian follicles is not only FSH dependent. FSH and LH have complementary roles in a natural cycle, especially in stimulating follicle growth and ovulation (4, 5). In assisted reproductive technology (ART) treatment cycles, when the long agonist protocol is used for controlled ovarian stimulation (COS), the agonist suppresses the circulating LH levels (6).

Profound suppression of LH concentrations in some normogonadotropic patients can cause several adverse effects like impairing adequate estradiol synthesis, fertilization rate, and the final clinical treatment outcome by increasing the risk of early pregnancy loss (7-10). In a study it was shown that the number of required FSH ampoules for ovarian stimulation was significantly higher in women receiving FSH alone comparing with patients who received human chorionic gonadotropine (HCG) to supplement LH activity (11). Also Lisi *et al* reported higher pregnancy rates in infertile patients treated with combined rFSH and rLH in comparison to control group. Another study showed that patients with high endogenous LH had higher implantation rates (12, 13).

In ART cycles, women undergoing ovarian stimulation can experience sever LH deficiency following over suppression of endogenous pituitary secretion due to Gonadotropin-releasing hormone (GnRH) analogues suppression, which in some patients degree of suppression is like hypo-hypo patients, so during ovarian stimulation some, not every patient requires exogenous LH replacement, because low levels of endogenous LH is insufficient for thecal cell function (14). 

The main clinical purpose of this study is to investigate the benefits from co-administration of rLH in normoresponder patients undergoing controlled ovarian hyper-stimulation for IVF or ICSI.

## Materials and methods

This prospective study was done with financial support of Yazd Research and Clinical Center for Infertility as a randomized single center clinical trial in 2012. The study was approved by the Ethics Committee of Infertility Center. Written informed consent was obtained from all patients prior to participate in the study. A total of 40 normoresponder patients were included in this study. The inclusion criteria were infertile women younger than 35 years old, day 3 FSH serum levels <10 U/L, male infertility or unexplained infertility, and body mass index (BMI) less than 30. 

**Figure 1:**
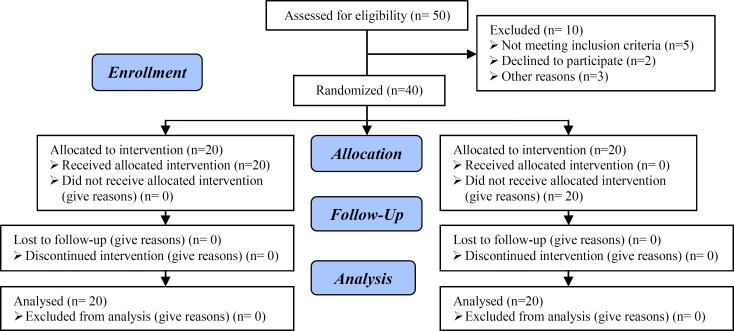
Consort flow diagram

Exclusion criteria were: azoospermia, uterine myoma, mild endometriosis, hydrosalpinx, history of previous IVF (successful or unsuccessful), history of endocrine diseases such as diabetes or thyroid disorders, and patients who had hysteroscopic surgery due to intrauterine lesions such as uterine sub-mucosal myoma or intrauterine adhesions (Figure 1). 

**Protocol**

Patients underwent pituitary down-regulation with Buserelin (Cinnafact ®, Laboratory, Cinnagen, Iran), using a daily dose of 500 mg, s.c., according to the long agonist protocol, starting on day 21 of the cycle preceding gonadotropine treatment and continued 250 mg/daily with start of menstruation until the day of hCG administration. Then treatment with r-hFSH alone (Gonal-F®, Laboratories Serono S.A., Aubonne, Switzerland) (75 IU per ampoule) was started on day 2 of menstruation with 2-3 ampoule/day based on ovarian response as assessed by sequential vaginal ultra sonography and serum estradiol measurement until the leading follicle had reached a diameter of 14 mm. Then patients were randomized to two groups of 20 patients, one group received r-hLH (lutropinalfa; Luveris®, Laboratories Serono S.A.), 75 IU s.c., for a maximum of 10 days (group 2) and the other group continued r-hFSH without r-hLH (group 1). 

Randomization was done with random numbers table. Ovulation was induced by administration of HCG (Profasi®, Laboratoires Serono S.A.), 10,000 IU i.m., when at least two or three follicles had reached a diameter of >17 mm. Endometrial thickness and estradiol levels were measured on HCG injection day. Transvaginal ultrasound-guided needle aspiration of oocytes was performed 34-36 h after HCG injection. IVF or ICSI were performed depending on the semen parameters. In both groups embryo transfer was done 2-3 days after follicular aspiration. The maturational status of oocytes and embryo grading (Hills 1998) was also performed. 

The embryos of highest morphological grade were transferred into the uterine cavity. Number of transferred embryos was determined based on patient's age, number and quality of embryos and up to 3 embryos per patients were transferred. For luteal phase support 100 mg/daily intramuscular injections of progesterone was administrated.14 days after embryo transfer serum β-hCG was checked. If it was positive, pregnancy would be approved. 3 weeks after positive βhCG, transvaginal sonography was done to confirm the presence of fetal sac and heart beat (clinical pregnancy). The results were compared in two groups. Luteal phase support was continued for 10 weeks of pregnancy. 

**Statistical analysis**

All statistical analysis was performed using SPSS software. Student’s *t*-test, Mann-Whitney *U*-test and χ^2^-test was used as appropriate. P-values below 0.05 were considered significant.

## Results

40 women fulfilling the criteria defined in materials and methods participated in this controlled trial study. Patient characteristics are shown in table I. The groups were comparable regarding patient characteristics. There were no significant differences among the groups regarding women’s age, basal LH and FSH, kind and cause of infertility. Details of ovarian stimulation and pregnancy outcomes in the two groups are summarized in tables II, III. Serum estradiol concentrations on the day of HCG administration were higher in the group receiving r-hFSH in addition to r-hLH in comparison to the group receiving FSH alone although they weren’t statistically significant. There weren’t significant differences in the stimulation duration, the number of gonadotropin ampoules, and endometrial thickness on day of HCG administration in two groups. 

Table III shows that supplementation with r-hLH has no influence on the total number of oocytes, mature oocytes and the total number of embryos. In relation to pregnancy rates, table III shows that implantation, chemical and clinical pregnancy rates were not significantly different between the two groups.

**Table I:** Demographic characteristics of the patients randomized to treatment

**Variables**		**Group 1 (r-hFSH alone)** **mean ± SD**	**Group 2 (r-hFSH+ r-hLH)** **mean ± SD**	**p-value** *
Number of patients		20	20	
Mean age of women (years)		31.35 ± 1.69	31.85 ± 1.59	0.34
Mean duration of infertility (years)		5.85± 2.35	6.15 ± 3.88	0.77
Basal LH (IU/l)		4.29±0.98	3.88 ± 0.73	0.13
Basal FSH (IU/l)		6.11±1.35	6.26 ± 1.25	0.71
Kind of infertility				
	Primary	20 (100%)	17 (85%)	
	Secondary	0 (0%)	3 (15%)	0.23
Cause of infertility				
	Male factor (%)	15 (75%)	12 (60%)	
	Tubal factor (%)	1 (5%)	5 (25%)	
	Unexplained (%)	4 (20%)	3 (15%)	0.23

* Independent t-test and chi square

**Table II:** Ovarian stimulation characteristics of the two treatment groups

**Variables**	**Group 1 (r-hFSH alone)** **mean ±SD**	**Group 2 (r-hFSH+ r-hLH)** **mean ±SD**	**p-value** *
Stimulation duration (days)	10.80 ± 1.32	10.70 ± 1.42	0.82
Ampoules of r-hFSH (75 IU)	23.20±7.14	25.95 ± 7.20	0.23
Endometrial thickness (mm)	8.05 ± 2.28	8.87 ± 1.45	0.18
Serum estradiol on HCG administration day (g/l)	1151 ± 853.01	2179 ± 2505.97	0.09

* Independent t-test

**Table III:** Pregnancy outcome characteristics of the two treatment groups

**Variables**	**Group 1 (r-hFSH alone)** **mean ± SD**	**Group 2 (r-hFSH+ r-hLH)** **mean ± SD**	**p-value** *
Number of oocytes obtained	8.25 ± 3.44	9.70 ± 4.44	0.77
Number of metaphase ΙΙ oocytes (MII)	7.15 ± 3.16	8.50 ± 4.37	0.70
Number of total embryos	4.00 ± 2.24	5.56 ± 3.75	0.84
Number of transferred embryos	2.35 ± 0.67	2.45 ± 0.60	0.15
Implantation rate (%)	5.8%	10%	0.58
Chemical pregnancy	3 (15%)	5 (25%)	0.69
Clinical pregnancy rate (%)	3 (15%)	5 (25%)	0.69
Miscarriage rate (%)	0	0	0

* Independent t-test and chi-square

## Discussion

In a natural cycle LH is essential to maintain adequate esteroidogenesis and follicular development. FSH and LH secretion by the pituitary controls normal follicular growth. These hormones are necessary for normal E_2_ biosynthesis as explained by two-cell, two-gonadotropin model (15, 16). LH is the initiator of many molecular events like up-regulation of LH and progesterone receptors expression in peri-ovulatory follicles (17-19). However increase in serum LH concentrations has been associated with impaired reproductive outcome in follicular phase (20-22). It is possible that in the late follicular phase, appearance of LH receptors on granulosa cells among maturating follicles, decrease FSH dependent development and promote equivalent responses of FSH and LH for continued follicular growth (23). 

It is widely shown that endogenous LH levels decrease and reach to the lowest levels during late follicular phase when FSH and GnRH agonist are used for ovarian stimulation. So, it seems logical that if LH supplementation has beneficial effects, late follicular phase would be the proper time for its administration (8, 24, 25).

This controlled clinical trial study compared efficacy of r-hLH co-administration with r-hFSH in the late follicular phase for ovulation induction in normoresponder patients who were undergoing ART treatment. Our result showed that r-hLH administration has no effect at critical time point of follicular development and in the exposure of low endogenous levels of LH due to the long agonist protocol in this age group of patients.

In this study the total number of obtained oocytes, mature oocytes, resulted embryos, the number of FSH ampoules, endometrial thickness, fertilization rate, embryo quality, implantation rate, chemical and clinical pregnancy rate were higher in r-hLH supplementation group, although they were not significantly different in two groups. It was indicated in a study by Lisi *et al* that rLH supplementation (75 IU/day) in 79 patients after down-regulation with triptorelin 0.1 mg, caused an increased pregnancy rate (12). Such differences in the implantation and clinical pregnancy rate should be interpreted with caution because it may suggest better results after rLH administration.

In present study stradiol levels on the day of hCG administration were higher in r-hLH supplementation group but it was not statistically significant. It is not clearly understood how stradiol effects follicle and oocyte development, although its role as growth factor is shown by different studies and evaluation of estradiol patterns is one of important markers of ART success (26-28). Also in a study by Marrs *et al *the addition of r-hLH had no significant effect on the number of MII oocytes and implantation rate in the study population. In women over 35 years implantation rate was significantly higher in rLH group, which suggests that older patients may benefit from LH supplementation (29). It should be noted that in our study the mean age of patients was 31 years which may be the cause of insignificant results.

Some recent studies have shown that FSH efficacy when co-administrated with rLH, is decreased which is reflected in increasing FSH vials needed for ovarian stimulation (6, 30, 31). Also in current study the number of needed FSH ampoules was higher in r-hLH administration group. Although in a recent meta-analysis 50 IU decease in FSH ampoules was seen when rLH was administrated (32). r-hLH supplementation was controversial for ovulation induction in ART patients (5, 33). In a study by Sills *et al *it was demonstrated that in a group of patients who received r-hFSH alone implantation and pregnancy rates were higher in comparison to the other group who received r-hFSH and supplementary r-hLH, although the differences were not statistically significant (34). In contrast in present study implantation and pregnancy rate were higher in r-hLH supplementation group, although it wasn’t significant. 

In a study by Tarlatzis *et al* the effect of rLH in late stimulation phase was evaluated after down regulation with long protocol. They found that rLH supplementation caused a higher E_2_ level on hCG administration day but it had no beneficial effect on ART outcome in young patients population (35). In summery according to our results r-hLH administration during ovulation induction doesn’t actually changes cycle performance, in normoresponder patients.

## Conflict of interest

The authors declare no conflict of interest regarding the relevant research and the present article.
